# Ultrasound reference values for peripheral nerve cross-sectional areas and indices in a sample of healthy individuals in Brazil

**DOI:** 10.1590/0100-3984.2022.0020

**Published:** 2022

**Authors:** Glauber Voltan, Fred Bernardes Filho, Helena Barbosa Lugão, Marcello Henrique Nogueira-Barbosa, Marco Andrey Cipriani Frade

**Affiliations:** 1 Dermatology Division, Department of Internal Medicine, Faculdade de Medicina de Ribeirão Preto da Universidade de São Paulo (FMRP-USP), Ribeirão Preto, SP, Brazil.; 2 Radiology Division, Department of Internal Medicine, Faculdade de Medicina de Ribeirão Preto da Universidade de São Paulo (FMRP-USP), Ribeirão Preto, SP, Brazil.

**Keywords:** Peripheral nerves/diagnostic imaging, Ultrasonography/methods, Reference values, Nervos periféricos/diagnóstico por imagem, Ultrassonografia, Valores de referência

## Abstract

**Objective:**

To establish peripheral nerve cross-sectional area (CSA) reference values (absolute values, measures of asymmetry, and measures of focality) for healthy individuals in Brazil.

**Materials and Methods:**

Sixty-six healthy volunteers underwent high-resolution ultrasound of the peripheral nerves. We obtained CSA measurements for three peripheral nerves, at specific locations: the median nerve, in the carpal tunnel (MT); the ulnar nerve, at the cubital tunnel site (UT) and at the pre-tunnel site (UPT); and the common fibular nerve, near the fibular head (FH). We calculated the CSA indices between the same sites on different sides (ΔCSAs) and between the ulnar nerve tunnel and pre-tunnel sites on the same side (ΔTPT).

**Results:**

A total of 132 neural sites were analyzed, and the following CSA values (mean ± SD, median) were obtained: MT (6.3 ± 1.9 mm^2^, 6.0 mm^2^); UT (6.2 ± 1.6 mm^2^, 6.1 mm^2^); UPT (5.6 ± 1.7 mm^2^, 5.4 mm^2^); and FH (10.0 ± 3.7 mm^2^, 9.9 mm^2^). The ΔCSA values (mean ± SD, median) were as follows: MT (0.85 ± 0.7 mm^2^, 0.95); UT (0.81 ± 0.62 mm^2^, 0.95); UPT (0.61 ± 0.51 mm^2^, 0.5); and FH (1.0 ± 0.77 mm^2^, 1.0). The ΔTPT (mean ± SD, median) was (1.0 ± 0.8 mm^2^, 1.0).

**Conclusion:**

Among individuals in Brazil, peripheral nerve CSA values tend to be higher among males and to increase with aging. However, the same does not appear to hold true for the ΔCSA or the ΔTPT, the exception being the difference between the right and left UT. Differences in CSA values greater than 2.5 mm^2^ between sides or between sites along the same nerve can indicate asymmetry or focal thickening in neuropathy, respectively.

## Abstract (pt)

**Objetivo:**

Estabelecer valores de referência da área de secção transversa (AST) dos nervos periféricos (valores absolutos e medidas de assimetria e de espessamento focal) para amostra de indivíduos brasileiros saudáveis.

**Materiais e Métodos:**

Sessenta e seis voluntários brasileiros saudáveis foram submetidos a ultrassonografia de alta resolução de nervos periféricos. As medidas da AST dos seguintes nervos periféricos foram obtidas em: mediano no túnel do carpo (MT), ulnar no túnel cubital (UT), pré-túnel ulnar (UPT) e fibular comum na cabeça da fíbula (FH). Os índices CSA foram obtidos entre os mesmos sítios em lados diferentes (ΔCSA) e entre os sítios distal e proximal do nervo ulnar (ΔTPT).

**Resultados:**

As seguintes médias ± desvio-padrão e mediana da AST foram obtidas para os 132 sítios dos nervos periféricos analisados: MT (6,3 ± 1,9 mm^2^; 6,0 mm^2^), UT (6,2 ± 1,6 mm^2^; 6,1 mm^2^), UPT (5,6 ± 1,7 mm^2^; 5,4 mm^2^) e FH (10,0 ± 3,7 mm^2^; 9,9 mm^2^). A média ± desvio-padrão e as respectivas medianas do ΔCSA em mm^2^ foram: 0,85 ± 0,7 [0,95] para MT, 0,81 ± 0,62 [0,95] para UT, 0,61 ± 0,51 [0,5] para UPT, 1,0 ± 0,77 [1] para FH, e 1,0 ± 0,8 [1,0] para ΔTPT.

**Conclusão:**

Os valores de AST tendem a ser maiores no sexo masculino, aumentando os valores absolutos das medianas das ASTs com o envelhecimento, mas não nos seus índices, ΔCSA e ΔTPT, exceto a diferença entre a AST dos nervos ulnares nos lados direito e esquerdo. Diferenças de valores de AST entre lados ou pontos no mesmo nervo maior que 2,5 mm^2^ podem significar neuropatia com assimetria e espessamento focal.

## INTRODUCTION

Peripheral nerves have traditionally been evaluated by a combination of anamnesis, physical examination, and electrophysiological study, methods that provide limited information about neural morphology. Electroneuromyography is an invasive, painful examination that provides data about neural function. High-resolution ultrasound provides information complementary to electroneuromyography data on the morphology of peripheral nerves, including the fascicular pattern and vascularization^(1)^. In addition, high-resolution ultrasound objectively assesses neural dimensions and locates possible compression zones within fibrous tunnels, tumors, and traumatic pathologies, as well as being a noninvasive, low-cost examination that is readily accessible and rapidly executed^(2,3,4)^, which makes it a reliable technique with high interobserver and intraobserver reliability^(2,5,6,7)^.

In unilateral neuropathies, the contralateral side can be used as an internal control^(8)^. In polyneuropathies, nerve enlargement, mainly identified though side-to-side comparisons, as well as through the evaluation of changes in the anteroposterior diameters of cross sections, the anteroposterior diameters of longitudinal views, and crosssectional areas (CSAs), has been well documented^(7,9,10,11)^. As detailed in Table 1, ratios between the CSAs of different segments of the same nerve—focality, tunnel versus pre-tunnel (ΔTPT), and intranerve variability—can also be evaluated, as can ratios between the CSAs of the same nerve on opposite sides of the body—right/left asymmetry, Δ right/left (ΔCSA), and side-to-side difference—with particular characteristics that can facilitate the differential diagnosis among multiple mononeuropathies, inflammatory polyneuropathies, and chronic demyelinating neuropathies**( 12,13)**. Although international studies have reported reference CSA values for the cervical nerve roots, as well as for the ulnar, median, radial, common fibular, tibial, and sural nerves, with good agreement between measurements, there are no well-established reference values for the Brazilian population^(13,14,15,16,17,18,19,20)^.

**Table 1:** Equations for calculating the difference between and the variability of peripheral nerve CSAs.

Variable	Approach	Equation
Asymmetry	Ratio	= [maximum CSA (right/left)] − [minimum CSA (right/left)]
	Difference	ΔMT = [maximum CSA MT (right/left)] − [minimum CSA MT (right/left)]
		ΔUT = [maximum CSA UT (right/left)] − [minimum CSA UT (right/left)]
		ΔUPT = [maximum CSA UPT (right/left)] − [minimum CSA UPT (right/left)]
		ΔFH = [maximum CSA FH (right/left)] − [minimum CSA FH (right/left)]
Focality	Ratio	= [maximum CSA (UT/UPT)] − [minimum CSA (UT/UPT)]
	Difference	ΔTPT = [maximum CSA (UT/UPT)] − [minimum CSA (UT/UPT)]

The objective of this study was to determine the absolute CSA values, the asymmetry indices (ΔCSA and CSA ratio), and the focality indices (ΔTPT and TPT ratio) for the peripheral nerves in a sample of healthy individuals in Brazil.

## MATERIALS AND METHODS

This study was approved by the Research Ethics Committee of the Clinical Hospital of the University of São Paulo at Ribeirão Preto School of Medicine and was performed in accordance with the Declaration of Helsinki. All participants gave written informed consent.

The minimum sample size calculated for the Brazilian population (211 million inhabitants), with a 95% confidence level and a 10% margin of error, was 97. Our sample comprised only 66 individuals. However, because the distribution of CSA was homogeneous between the right and left nerves, 132 neural sites were considered for statistical analysis, which makes ours the largest sample among the studies evaluated and meets the sample size calculation criteria. The calculation (performed at https://calculareconverter.com.br/calculo-amostral/ and verified at https://solvis.com.br/calculos-de-amostragem/) showed that 132 neural sites in a population of 211 million, with a 95% CI, would have a margin of error of 8.53%.

For this cross-sectional study, we recruited 85 individuals without peripheral neuropathy from health care facilities in the southeastern, northern, and northeastern regions of Brazil. Individuals with neurological symptoms (loss of strength, paresthesia, electric shock-like pain, pain, or cramps) were excluded, as were those with a body mass index ≥ 35.0 kg/m^2^, those diagnosed with a metabolic disease or peripheral neuropathy, and those who had had a limb amputated.

### Peripheral nerve ultrasound

**Figure 1:**
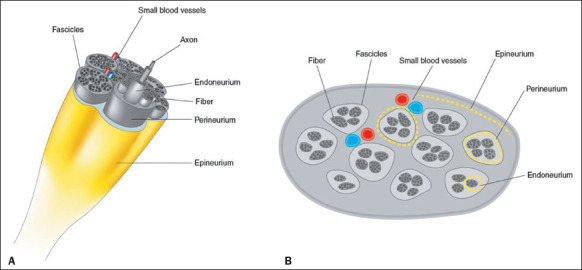
Schematic representations of a normal peripheral nerve and its morphological structures. Three-dimensional view (A) and high-resolution ultrasound cross-sectional view (B), showing the honeycomb pattern.

**Figure 2:**
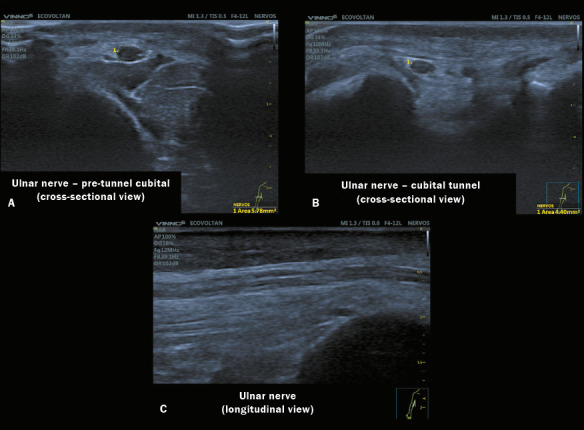
High-resolution neuromuscular ultrasound images of three sites of the same ulnar nerve obtained with a 4–17 MHz transducer in a VINNO 6 system. A: Image of the ulnar nerve with a normal CSA at the proximal (pre-tunnel) site, showing preserved echogenicity and fascicular pattern. B: Image of a normal ulnar nerve within the cubital tunnel in the right arm. C: Image of the ulnar nerve in a longitudinal view.

Between 2015 and 2019, two radiologists, each with more than three years of experience in musculoskeletal and neuromuscular ultrasound, used a fixed ultrasound system with a 5–12 MHz linear transducer (HD-11; Philips Medical Systems, Best, The Netherlands) to evaluate 36 healthy individuals. The same two radiologists evaluated another 15 healthy individuals by using a portable ultrasound system with a 4–16 MHz linear transducer (HM70; Samsung Medison Co., Ltd., Seoul, South Korea) and evaluated an additional 15 healthy individuals by using a portable ultrasound system with a 4–17 MHz linear transducer (VINNO 6; VINNO Technology, Suzhou, China). As shown in the schematic representation of a peripheral nerve and its morphological structures (Figure 1), the short and long axes of each nerve were scanned bilaterally, as depicted in Figure 2, and the CSAs were obtained by holding the transducer perpendicular to the surface of the nerve being scanned, without putting any pressure on the structures. The neural sites assessed were defined according to their proximity to bony landmarks, increasing the reproducibility of the method by being well-established sites for neural compression or common sites for electrophysiological assessment. The CSA was determined at those sites with a continuous scan, internally to the hyperechoic borders of the epineurium. Three consecutive measurements were made at each site, and the average of the three measurements was used in the analysis. To detect asymmetry, we evaluated three nerves, bilaterally, at four sites, as recommended by Frade et al.^(16)^: the median nerve, in the carpal tunnel (MT); the ulnar nerve, at the cubital tunnel site (UT) and at the pretunnel site (UPT, defined as a point 3–5 cm above the medial epicondyle of the humerus); and the common fibular nerve near the fibular head (FH).

### Systematic review

**Figure 3:**
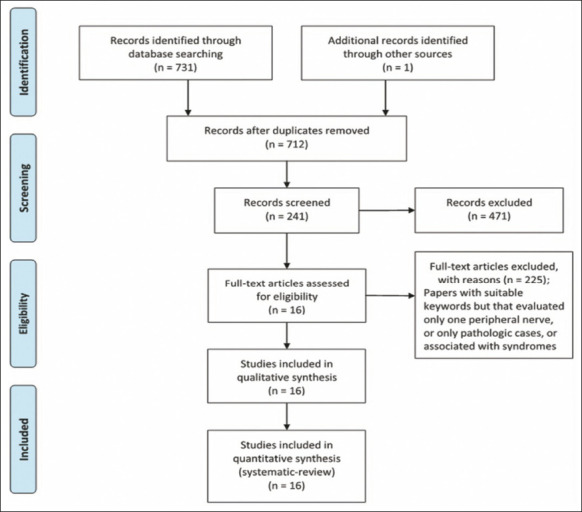
Flow chart of the selection of articles for the systematic review

In the year 2020, we organized a systematic review in accordance with the methods recommended in the Preferred Reporting Items for Systematic Reviews and Meta-Analyses (Figure 3). Searches for articles in English or Portuguese were carried out in the PubMed, Embase, and Virtual Health Library databases, using the following search string: peripheral nerves AND ultrasound AND CSA OR reference values OR normal values. Studies that evaluated cranial nerves were excluded, as were those that did not include normal or reference values, those that addressed peripheral nerve pathologies exclusively (i.e., did not include healthy individuals), those that did not clearly describe the anatomical site(s) evaluated, and those that evaluated only one neural site (e.g., the median nerve in the carpal tunnel). One study, conducted by Won et al.^(18)^, was included despite not matching the keywords chosen, because it was cited in other articles as a study that had assessed the peripheral nerves of the upper limbs. Therefore, a total of 16 studies were selected^(2,8,10,13,14,16,18,20,21,22,23,24,25,26,27,28)^.

### Statistical analysis

The CSA values were compiled in the Microsoft Excel program, and statistical analyses were performed with GraphPad Prism software, version 8 (GraphPad Software Inc., San Diego, CA, USA). The CSA values (in mm^2^) are expressed as means with standard deviations and medians for each of the eight sites assessed (four on each right and left side). Asymmetries were detected by using paired t-tests. The CSA measurements were used in order to calculate the following indices^(16)^: the differential CSA index (ΔCSA), determined by calculating the difference between the minimum and maximum CSA measurements for each neural site independent of the side; and the ulnar nerve tunnel/pre-tunnel CSA differential (ΔTPT), calculated on one side and defined as the difference between the CSA of the UPT and that of the UT. The following ratios were also calculated: the CSA ratio, calculated by dividing the maximum CSA by the minimum CSA for each neural site independent of the side; and the ulnar TPT ratio, calculated by dividing the maximum CSA by the minimum CSA for the UPT and UT sites along the ulnar nerve on the same side^(12,13)^.

Using Microsoft Excel, we constructed a database to record the following variables, from the present study and from the studies included in the review: the means (with standard deviations) and medians for CSA and ΔCSA; the number of subjects assessed; the sex of the subjects assessed; the age of the subjects assessed; and the device(s) employed. All of the data extracted from the articles were compared with those obtained in the present study. Differences in sample size and in the mean values were calculated by using t-tests for independent samples with unequal variances, in SciPy 1.8.0 for Python.

## RESULTS

We selected 66 healthy volunteers (28 men and 38 women) for inclusion in the study. The mean age of the individuals in the sample was 27 ± 13 years (median, 25 years; range, 7–60 years). As calculated from high-resolution ultrasound imaging, the mean and median CSAs for the neural sites on the right/left sides were as follows: 6.4 ± 1.9/6.2 ± 1.9 and 6.0/5.9, respectively, for the MT (*p* = 0.05); 6.2 ± 1.5/6.3 ± 1.7 and 6.5/6.0, respectively, for the UT (*p* > 0.05); 5.6 ± 1.7/5.5 ± 1.7 and 5.3/5.5, respectively, for the UPT (*p* > 0.05); and 10.2 ± 3.6/9.8 ± 3.8 and 10.1/9.0, respectively, for the FH (*p* > 0.05).

Table 2 shows the CSA, ΔCSA, CSA ratio, and ΔTPT values, by subject age range, for the 132 neural sites evaluated in the present study. We found no significant differences between the right and left sides in terms of the CSA for any of the neural sites.

**Table 2:** Distribution of ultrasound indices (CSA, ΔCSA, ΔTPT, CSA ratio, and TPT ratio) by age range and upper limit.

Variables	Age range (years)					Upper limit (mean + 2 SD)
	7–14	15–30	31–45	46–60	15–60	
Sex						
Male, n	6	12	4	6	22	
Female, n	11	12	11	4	27	
Total (R + L), n	34	48	30	20	98	
Overall CSA, mean ± SD [median]	11.7 ± 2.1 [12.0]	22.9 ± 4.6 [23.0]	37.0 ± 4.6 [36.0]	49.6 ± 2.5 [49.0]	27 ± 13 [25.0]	
CSA (mm^2^), mean ± SD [median]						
MT	5.5 ± 1.7 [5.0]	6.7 ± 2.0 [6.0]	6.8 ± 2.0 [6.2]	6.4 ± 1.2 [6.3]	6.6 ± 1.9 [6.0]	10.4
UT	5.2 ± 1.4 [6.0]	6.6 ± 1.7 [6.2]	6.6 ± 1.5 [6.7]	6.7 ± 1.0 [7.0]	6.7 ± 1.5 [6.1]	9.7
UPT	4.6 ± 1.2 [5.0]	6.3 ± 2.1 [5.8]	5.7±1.3 [5.6]	6.0±1.2 [6.3]	6.0 ± 1.7 [5.4]	9.4
FH	8.5 ± 3.0 [8.6]	10.7 ± 3.4 [11.0]	10.6 ± 4.8 [10.5]	10.9±2.9[10]	10.7 ± 3.8 [9.9]	18.3
∆CSA (mm^2^), mean ± SD [median]						
MT	0.84 ± 0.6 [0.9]	0.6 ± 0.7 [0.4]	1.0 ± 0.7 [1.0]	0.9±0.8 [0.8]	0.8 ± 0.7 [0.9]	2.2
UT	0.6 ± 0.5 [1.0]	1.0 ± 0.7 [1.0]	0.8 ± 0.5 [0.9]	0.8±0.6 [0.7]	0.9 ± 0.6 [0.9]	3.1
UPT	0.6 ± 0.6 [0.3]	0.7 ± 0.4 [0.5]	0.6 ± 0.6 [0.4]	0.8±0.6 [0.5]	0.6 ± 0.4 [0.5]	1.4
FH	1.2 ± 0.9 [1.0]	0.9 ± 0.7 [0.75]	0.9 ± 0.6 [0.8]	1.1±0.8 [1.0]	0.9 ± 0.7 [1.0]	2.3
∆TPT (mm^2^), mean ± SD [median]	1.0 ±0.8 [1.0]	0.9 ± 0.8 [0.65]	1.2 ± 0.8 [1.1]	0.7±0.6 [0.6]	1.0 ± 0.8 [1.0]	2.6
CSA ratio, mean ± SD [median]						
MT	1.2 ± 0.3 [1.2]	1.1 ± 0.1 [1.1]	1.2 ± 0.1 [1.1]	1.2±0.1 [1.1]	1.1 ± 0.14 [1.1]	1.38
UT	1.1 ± 0.1 [1.0]	1.2 ± 0.2 [1.2]	1.1 ± 0.1 [1.1]	1.1±0.1 [1.1]	1.1 ± 0.12 [1.1]	1.34
UPT	1.2 ± 0.2 [1.0]	1.1 ± 0.1 [1.1]	1.1 ± 0.1 [1.1]	1.0±0.0 [1.0]	1.13 ± 0.14 [1]	1.41
FH	1.2 ± 0.1 [1.1]	1.1 ± 0.1 [1.1]	1.1 ± 0.1 [1.1]	1.1±0.1 [1.0]	1.1 ± 0.10 [1.1]	1.3
TPT ratio, mean ± SD [median]	1.2 ± 0.2 [1.2]	1.2 ± 0.2 [1.1]	1.2 ± 0.2 [1.2]	1.1±0.2 [1.0]	1.2 ± 0.19 [1.2]	1.58

R, right; L, left; SD, standard deviation.

**Figure 4:**
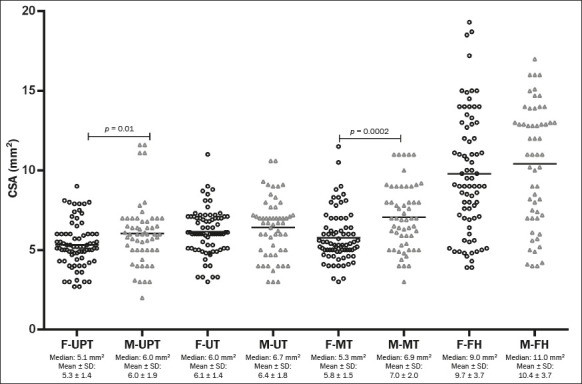
Column chart showing the distribution of CSA values (mm^2^) for the neural sites evaluated, by sex. The individual means and standard deviations of the absolute CSA values at the UPT, UT, MT, and FH sites were compared between the sexes by nonparametric and unpaired analysis with the Mann-Whitney test. The data show a trend toward higher values for men, although the difference was significant only for the UPT and MT. F, female; M, male; SD, standard deviation.

Absolute peripheral nerve CSA values were stratified by sex to analyze the differences (Figure 4). The data show a trend toward higher values in men, although that difference was significant only for the UPT and MT.

Except for those obtained for the UPT, the CSA values increased with advancing age, being lowest in the 7- to 14-year age group (*p* < 0.05). With the exception of a significant difference in CSA between the right and left UT (*p* < 0.05), the CSA indices (ΔCSA and ΔTPT) did not differ with advancing age. As can be seen in Table 2, the CSA ratio and TPT ratio were similar in all age groups.

In view of the paucity of data in the literature regarding asymmetry and focality, we decided to perform a comparative analysis of the minimum and maximum CSA values calculated by determining the difference between the two and those calculated by determining the ratio between the two. Frade et al.^(16)^ analyzed the side-to-side difference between the minimum and maximum CSA values at the same site (internerve ΔCSA) and between two sites along the same ulnar nerve (intranerve ΔTPT), whereas Kerasnoudis et al.^(13)^, Tagliafico et al.^(5,8)^, and Padua et al.^(12)^ assessed the side-to-side relationship/ratio between minimum and maximum CSA measurements (internerve ΔCSA) and between two sites along the same ulnar nerve (intranerve ΔTPT). Given those differences in asymmetry and focality, our objective was to evaluate the values calculated by both methods, considering the difference and the ratio for each of the sites analyzed (Table 3).

**Table 3:** Indices calculated for the neural sites evaluated, in the present study and in four of the studies included in the systematic review of the literature, by approach.

Parameter	Current study		Frade et al.^(16)^	Kerasnoudis et al.^(13)^	Won et al.^(18)^	Qrimli et al.^(2)^
	Difference (∆)	Ratio	Difference (∆)	Ratio	Ratio	Difference, ∆
Equation	Maximum (R or L) − minimum (R or L)	Maximum (R or L) / minimum (R or L)	Maximum (R or L) − minimum (R or L)	Side-to-side R/L	R − L / (< R or L) × 100	Upper limit side-to-side
N	66	66	46 or 48*	75	97	100
Age range (years)	15–60	15–60	≥ 18	≥ 18	20–69	≥ 18
CSA, mean ± SD						
MT	0.85 ± 0.7	1.1 ± 0.14	1.0 ± 0.8	1.21 ± 0.04	—	3.3
UT	0.81 ± 0.62	1.1 ± 0.12	1.0 ± 0.7	1.2 ± 0.25	1.31 ± 0.25	2.5
UPT	0.61 ± 0.51	1.13 ± 0.14	0.9 ± 0.7	—	1.25 ± 0.2	2.5
FH	1.0 ± 0.77	1.1 ± 0.10	1.1 ± 1.1	1.19 ± 0.23	—	4.3
Ulnar TPT, mean ± SD	1.0 ± 0.81	1.2 ± 0.19	1.4 ± 1.1	1.5 ± 0.5	—	—

* 46 for the UT and UPT; 48 for the MT and FH.

R, right; L, left.

To assess the differences between our measurements and those reported in the literature, we performed a comparative analysis considering sample size and mean values with standard deviations (Table 4). Our CSA values were similar to those reported in the studies conducted by Frade et al.^(16)^ and Druzhinin et al.^(26)^ for the MT (*p* > 0.05); to those reported in the studies conducted by Cartwright et al.^(14)^, Druzhinin D et al.^(26)^, and Lothet et al.^(29)^ for the UT (*p* > 0.05); to those reported in the studies conducted by Frade et al.^(16)^ and Won et al.^(18)^ for the UPT (*p* > 0.05); and to only those reported in the study conducted by Tagliafico et al.^(8)^ for the FH (*p* > 0.05).

**Table 4:** Comparative analysis between the CSA values obtained in the current study and those reported in 13 of the studies included in the systematic review of the literature, by neural site.

Reference	Neural site															
	MT				UT				UPT				FH			
	Nerves (n)	Mean	SD	*P* *	Nerves (n)	Mean	SD	*P* *	Nerves (n)	Mean	SD	*P* *	Nerves (n)	Mean	SD	*P* *
Lothet et al.^(29)^	50	10	2.6	< 0.001	30	8.2	1.3	0.8	—	—	—	—	60	11.2	3.3	0.03
Bedewi et al.^(27)^	138	9.8	2.8	< 0.001	138	7.5	2.3	< 0.001	138	7.6	2.6	< 0.001	138	8.9	3.2	0.006
Druzhinin et al.^(26)^	44†	6.58	1.97	0.403	44†	5.78	1.9	0.153	44†	6.41	2.1	0.011	44†	7.82	2.2	< 0.001
Kerasnoudis et al.^(13)^	150	8	2	< 0.001	—	—	—	—	—	—	—	—	—	—	—	—
Cartwright et al.^(21)^	100	9.8	2.4	< 0.001	—	—	—	—	—	—	—	—	—	—	—	—
Boehm et al.^(10)^	56	8.5	1.8	< 0.001	56	7.6	2.1	< 0.001	56	6.3	1.6	0.009	56	8.9	2	0.037
Grimm et al.^(24)^	100‡	10.6	2.9	< 0.001	100‡	8.7	2.9	< 0.001	100‡	7	1.2	< 0.001	100‡	8.4	1.6	< 0.001
Frade et al.^(16)^	96	5.9	1.5	0.09	92	6.7	2.2	0.05	92	5.9	1.8	0.21	96	8.2	4.4	0.001
Qrimli et al.^(2)^	100‡	10.2	2.4	< 0.001	100‡	6.9	2.3	0.007	100‡	6.8	2.3	< 0.001	100‡	11.1	3.5	0.023
Won et al.^(18)^	194	8.3	1.5	< 0.001	—	—	—	—	194	5.8	1	0.18	—	—	—	—
Cartwright et al.^(20)^	—	—	—	—	—	—	—	—	—	—	—	—	120	11.2	3.3	0.007
Seok et al.^(23)^	—	—	—	—	—	—	—	—	—	—	—	—	94‡	9.2	2.9	0.082
Tagliafico et al.^(32)^	80	8.2	2.3	< 0.001	80	5.9	3.0	0.344	—	—	—	—	120	11.4	8	0.072
Current study	132	6.3	1.9	—	132	6.2	1.6	—	132	5.6	1.7	—	132	10	3.7	—

* Results of the present study versus those of the studies included in the systematic review.

† The authors considered only individuals ≥ 15 years of age (n = 22).

‡ The authors considered only the right side for neural site measurements; therefore, the number of individuals was equal to the number of nerves evaluated.

Regarding the measurements of internerve asymmetry, intranerve asymmetry, and focality (Table 3), the indices calculated between CSA measurements were reported as the differences between the minimum and maximum values in four of the studies evaluated^(2,16,18,23)^, whereas they were reported as the ratio between those values in two^(8,13)^.

## DISCUSSION

High-resolution ultrasound has proven to be an excellent diagnostic method for peripheral nerve assessment, providing information about echotexture and fascicular patterns in neuropathies, mainly by CSA measurements**( 30)**. An increased CSA permits precise localization in compressive neuropathies and in neural tumors^(3,31)^. The localization of neural thickening, such as at the UPT, can facilitate the diagnosis of leprosy neuropathy^(16,33)^. The thickening of multiple nerves has also been reported in acquired and hereditary polyneuropathies^(9,11,34)^.

We compared our results with those of the other studies evaluated, in terms of the mean values obtained and the size of the independent samples. Our results are similar to those reported by Druzhinin et al.^(26)^ and Frade et al.^(16)^ for the MT; to those reported by Lothet et al.^(29)^, Druzhinin et al.^(26)^, and Cartwright et al.^(14)^ for the UT; to those reported by Frade et al.^(16)^, Won et al.^(18)^, and more recently by Bae et al.^(22)^ for the UPT; and only to those reported by Tagliafico et al.^(8)^ for the FH. The fact that there were so few similarities between our results and those of the other studies is likely due to differences in the populations evaluated and in the methodologies employed.

In the present study, the CSA indices showed little variation, the maximum upper limit for the ΔCSA between the right and left sides being 2.0 mm^2^. The equation involving subtraction rather than division or percent index proved to be more practical for daily use, as well as being more highly recommended for the calculation of differences with smaller detectable values, possibly indicating earlier changes in variability.

The CSA reference values for the MT were similar among the subjects < 15 years of age and lower among those ≥ 15 years of age; for the UT, they were higher among the subjects < 15 years of age and similar among those ≥ 15 years of age; and for the FH, they were slightly higher among the subjects < 15 years of age and similar among those ≥ 15 years of age.

Our findings for the UPT and MT, together with those reported in the literature, indicate that CSA values tend to be higher among males, as well as showing that the median of the absolute CSA values increases with aging. The differences between the 7- to 14-year age group and the three other age groups evaluated were significant for the UPT and MT. In the overall age range (≥ 15 years), the values obtained in the present study differed from those reported in the literature for most of the sites assessed^(2,8,16,18,23,24,27,29,32,35,36)^, with small variations in the means and standard deviations, as well as CSA differences of up to 2.0 mm^2^, in the articles reviewed; the exception was the MT, for which the values in the literature were higher than those obtained for our sample. However, for most of the neural sites evaluated, the values in the literature were significantly higher than those obtained for our sample, which underscores the importance of studying specific populations like the one evaluated in the present study.

Calculating the indices between the CSA values by determining the difference between the minimum and maximum values seem to be a better way to identify asymmetries, as well as to diagnose and monitor neuropathy. In contrast, calculating those indices by determining the ratio between the minimum and maximum values maintains the proportionality but might not indicate abnormalities or might delay their detection when there is thickening at both neural sites.

Among the neural sites analyzed in our study, the asymmetry index ranged from 1.4 mm^2^ to 3.1 mm^2^ and the focality index varied by 2.6 mm^2^. To simplify the analysis, we hypothesized that 2.5 mm^2^ could be used as the upper limit of the indices proposed for asymmetry and focal thickening. That value is in agreement with the cutoff values cited in the literature, except for that cited in one study evaluating the ulnar nerve^(2)^ and that cited in another study evaluating the common fibular nerve^(23)^. However, that does not mean that we can use these cutoffs in clinical practice to differentiate between normal nerves and nerves affected by neuropathy, given that our sample did not include patients with peripheral neuropathy. Nevertheless, if values above 2.5 mm^2^ are found for both indices, there is a need for a careful assessment, including close inspection of the fascicular pattern and echogenicity.

Our study has some limitations. The ultrasound measurements were made by two different observers using different equipment and transducers, which could have resulted in heterogeneity of the results. In addition, we did not evaluate interobserver variability. Furthermore, the sample was relatively small, especially when stratified by age. However, none of those limitations had any negative impact on our analysis or on the conclusions drawn.

## CONCLUSION

In this study, we have demonstrated that the peripheral nerve CSAs in a sample of the Brazilian population were lower than most of those reported in the literature. On the basis of our findings, we established the following reference values for the Brazilian population: 6.3 ± 1.9 mm^2^ for the MT; 6.2 ± 1.6 mm^2^ for the UT; 5.6 ± 1.7 mm^2^ for the UPT; and 10 ± 3.7 mm^2^ for the FH. High-resolution ultrasound proved to be an important diagnostic tool for peripheral nerve assessment, especially for the quantitative evaluation of CSAs and the respective indices. Values higher than 2.5 mm^2^ might indicate the need for a careful investigation to identify peripheral neuropathy (asymmetric or focal). The combination of nerve enlargement, as determined by CSA, and indices above the upper limit can facilitate the classification and characterization of the distribution of neuropathies (hypertrophic, asymmetric, or focal), as well as their differential diagnosis.
